# Carer administration of as-needed subcutaneous medication for breakthrough symptoms in people dying at home: the CARiAD feasibility RCT

**DOI:** 10.1136/bmjopen-2024-084476

**Published:** 2025-06-12

**Authors:** Marlise Poolman, Stella Wright, Annie Hendry, Nia Goulden, Emily Holmes, Anthony Byrne, Paul Perkins, Zoe Hoare, Annmarie Nelson, Julia Hiscock, Dyfrig A Hughes, Julie O’Connor, Betty Foster, Liz Reymond, Penney Lewis, Bee Wee, Rossella Roberts, Anne Parkinson, Sian Roberts, Clare Wilkinson

**Affiliations:** 1 North Wales Centre for Primary Care Research, Bangor University, Wrexham, UK; 2 Specialist Palliative Care, Betsi Cadwaladr University Health Board, Wrexham, UK; 3 Bangor University, Bangor, UK; 4 NWCPCR, Bangor University, Wrexham, UK; 5 North Wales Organisation for Randomised Trials in Health, Bangor University, Bangor, UK; 6 Marie Curie Research Centre, Cardiff University, Cardiff, UK; 7 Gloucestershire Hospitals NHS Foundation Trust, Cheltenham, UK; 8 Sue Ryder Leckhampton Court, Cheltenham, UK; 9 School of Medicine, Cardiff University College of Biomedical and Life Sciences, Cardiff, UK; 10 Centre for Health Economics and Medicines Evaluation, Bangor University, Bangor, UK; 11 Patient and Public Involvement, Bangor, UK; 12 Patient and Public Involvement, North Wales Cancer Patient Forum, Bodelwyddan, UK; 13 Brisbane South Palliative Care Collaboration, Griffith University, Nathan, Queensland, Australia; 14 Centre of Medical Law and Ethics, Dickson Poon School of Law, King’s College London, London, UK; 15 Palliative Medicine, University of Oxford, Oxford, UK; 16 School of Psychology, Bangor University, Bangor, UK; 17 Leckhampton Court Hospice, Cheltenham, UK; 18 Betsi Cadwaladr University Health Board, Bangor, UK

**Keywords:** PALLIATIVE CARE, Caregivers, Adult palliative care

## Abstract

**Objectives:**

To determine if carer administration of as-needed subcutaneous medication for common breakthrough symptoms in people dying at home is feasible and acceptable in the UK, and if it would be feasible to test this intervention in a future definitive randomised controlled trial.

**Design:**

We conducted a two-arm, parallel-group, individually randomised, open pilot trial of the intervention versus usual care, with a 1:1 allocation ratio, using convergent mixed methods.

**Setting:**

Home-based care without 24/7 paid care provision, in three UK sites.

**Participants:**

Participants were dyads of adult patients and carers: patients in the last weeks of their life who wished to die at home and lay carers who were willing to be trained to give subcutaneous medication. Strict risk assessment criteria needed to be met before the approach, including a known history of substance abuse or carer ability to be trained to competency.

**Intervention:**

Intervention-group carers received training by local nurses using a manualised training package.

**Primary outcome measures:**

Quantitative data were collected at baseline and 6–8 weeks post-bereavement and via carer diaries. Interviews with carers and healthcare professionals explored attitudes to, experiences of and preferences for giving subcutaneous medication and experience of trial processes. The main outcomes of interest were feasibility, acceptability, recruitment rates, attrition and selection of the most appropriate outcome measures.

**Secondary outcome measures:**

The secondary outcome measure was time to symptom relief, calculated using data items from the carer diary, after the patient had died.

**Results:**

In total, 40 out of 101 eligible dyads were recruited (39.6%), which met the feasibility criterion of recruiting >30% of eligible dyads. The expected recruitment target (≈50 dyads) was not reached, as fewer than expected participants were identified. Although the overall retention rate was 55% (22/40), this was substantially unbalanced (30% (6/20) usual care and 80% (16/20) intervention). The feasibility criterion of >40% retention was, therefore, considered not met. A total of 12 carers (intervention, n=10; usual care, n=2) and 20 healthcare professionals were interviewed. The intervention was considered acceptable, feasible and safe in the small study population. The intervention group had a considerably shorter time to medication administration than the usual-care group (median time to administer medication in intervention=5 min, usual-care=105 min). Intervention group carers felt confident in administering medication. Healthcare professional support was sought by intervention group carers in 24 out of 147 (16.3%) medication administration entries. The context of the feasibility study was not ideal, as district nurses were overstretched, unfamiliar with research methods and possibly not in equipoise. A disparity in readiness to consider the intervention was demonstrated between carers, who were uniformly enthusiastic, and healthcare professionals who were not. Findings confirmed methodological and ethics issues pertaining to researching the last days of life care.

**Conclusion:**

The success of a future definitive trial is uncertain because of equivocal results in the progression criteria, particularly poor recruitment overall and a low retention rate in the usual-care group. Future work regarding the intervention should include understanding the context of UK areas where this has been adopted, ascertaining wider public views and exploring healthcare professional views on burden and risk in the NHS context. There should be consideration of the need for national policy and the most appropriate quantitative outcome measures to use. This will help to ascertain if there are unanswered questions to be studied in a trial.

**Trial registration number:**

ISRCTN11211024.

**Strengths and limitations of this study**

- The use of both quantitative and qualitative methodologies is a strength of this study.
- Strong public contribution throughout all stages of the research is a strength of this study.
- A limitation to this study is that a series of progression criteria were not met.
- Differential recruitment to the control group is a limitation of this work.

## Introduction

 Caring for the dying during their last few days of life in a place of their preference is an essential part of health and social care. The majority of people express a wish to die at home (79%), but only half of those achieve this.^1^ The likelihood of patients remaining at home often depends on the availability of able and willing informal carers,^2 3^ who take on numerous care tasks. Extending the role of carers to include administering subcutaneous injections has proven key in achieving better symptom control for those dying at home in other countries.^4^

Pain, nausea/vomiting, restlessness/agitation and noisy breathing (rattle) are common symptoms in people who are dying.^5 6^ In addition to regular (background) medication, given via continuous subcutaneous infusion using a syringe pump, guidelines suggest using additional (‘as-needed’) medication for symptoms that ‘break through’.^7 8^ As dying patients are commonly unable to take oral medication, as-needed medication is most often given as a subcutaneous injection by a healthcare professional (HCP), in the UK usually a district nurse.

Medication for breakthrough symptoms is usually prescribed in advance (anticipatory prescribing) and kept in the patient’s home.^9 10^ Medication administration can be severely delayed by HCPs’ travel time to the patients’ home and/or the non-availability of anticipatory medication in the home. Delays happen even with dedicated out-of-hours ‘rapid response’ nursing services for patients dying at home. Breakthrough pain is usually quick in onset and has a median duration of 30 min.^11^ Long waits mean that pain is often not adequately managed in the home setting, as shown in the National Survey of Bereaved People (VOICES).^1^

This study focused on the timely administration of as-needed medication for dying patients who were being cared for at home.

### Objectives

The research question was ‘Is carer administration of as-needed subcutaneous medication for common breakthrough symptoms in people dying at home feasible and acceptable in the UK, and is it feasible to test this intervention in a future definitive randomised controlled trial (RCT)?’.

- P (People)=people in their last days of life who are being cared for at home, and their carers.
- I (Intervention)=carer administration of as-needed subcutaneous medication for common breakthrough symptoms (pain, restlessness/agitation, nausea/vomiting and noisy breathing), supported by tailored education.
- C (Control)=usual care (HCP administration of as-needed subcutaneous medication).
- O (Outcome)=main outcomes of interest–feasibility and acceptability of the trial and intervention, recruitment, attrition and contamination.

Based on a rapid literature review and to inform the design of a phase III trial, we aimed to:

- Adapt a successful Australian intervention as a standardised, manualised intervention for UK carer administration of as-needed subcutaneous medication for breakthrough symptoms in patients dying at home.^4 12^
- Establish the feasibility of the trial and the intervention by assessing acceptability, ability to recruit, attrition rates and suitability to a UK context. This was achieved by conducting an external randomised pilot trial with an embedded qualitative component.
- Identify attributes pertinent to carer’s preferences for HCP versus own administration of as-needed subcutaneous medications for patients dying at home (as part of qualitative component) and to establish the feasibility of completion of the Carer Experience Scale (assessed in the pilot trial).^13^

## Methods

Expert consensus workshops were conducted in the planned recruitment areas to map current processes and to gain a clearer understanding of how the intervention could be delivered in the local NHS context.^14^ These half-day, face-to-face workshops were attended by patients, carers, general practitioners, district nurses, pharmacists, specialist palliative care clinicians and research nurses, and informed the development of the trial processes and materials.

An existing well-established Australian manualised training package was reviewed and adapted for use in the trial through discussion with the Australian authors,^12 15^ input from the expert consensus workshops and consideration of the UK context.

### Feasibility RCT

#### Design

A multi-centre, two-arm, parallel-group, individually randomised, open pilot trial of the intervention versus usual care, with a 1:1 allocation ratio, using convergent mixed methods was conducted.^16^ Further details can be found in the study protocol.^14^

#### Setting

Home-based care without 24/7 paid care provision, in three UK sites (North Wales, Vale of Glamorgan and Gloucestershire). A 12-month recruitment period per site was planned.

#### Participants

Participants were dyads of adult patients and carers. Dyads consisted of an adult (ie, aged ≥18 years) patient in the last weeks of their life who was likely to lose the oral route of administration for medication and who had expressed a preference to die at home and their adult (unpaid) lay/family carer, also aged over 18, who was willing to take on this extended role and receive subcutaneous injection training.

Prognostication was reliant on the professional judgement of and agreement among the attending HCP team (ie, clinical estimate of survival). There was an assumption that the carer would spend a significant amount of time with the patient. When more than one carer was available, we asked the patient to identify which carer they would like to be included in the study.

A dyad was excluded if any one of the following criteria was met: safety concerns (eg, drug allergies or the ability of the carer to carry out required tasks) OR relational concerns OR misuse of drugs concerns OR objections to the concept of lay carer administration OR lack of access to, or willingness to engage with, healthcare support systems.

#### Trial procedures

Study recruitment and data collection procedures are summarised in table 1.

**Table 1:** Schedule of study procedures

Procedures	Time point			
	Screening	Baseline	Study period(last days of life)	Post-bereavement
Eligibility assessment	As per dyad inclusion/exclusion criteria	As per dyad inclusion/exclusion criteria	As per dyad inclusion/exclusion criteria	
Informed consent	Advance consent from dyad	Check consent	Check consent (personal consultee assent might be required when patient loses capacity)	
Demographics		CRF		
Medical history		CRF		
Concomitant medication		CRF	CRF	
Randomisation				
Assessment: symptom control				
Symptom scores			Tool: *carer diary* Completed by: carer When: at every occurrence of symptom	
Overall symptom burden				Tool: *Family MSAS-GDI* Completed by: carer When: at post-bereavement visit Tool: *Qualitative interviewing* Completed by: carer When: post-bereavement
Time to symptom relief			Measure: *episodes resolved in 30 min* Completed by: carer When: 30 min after drug administration Measure: *time when control achieved or symptom reduced to an acceptable level* Completed by: carer When: after drug administration	Tool: *Qualitative interviewing* Completed by: carer When: post-bereavement
Assessment: safety				
RA tool	Tool: *adapted tool based on Fuller’s self-medication risk assessment screening tool* Completed by: HCP When: prior to dyads being approached to take part in the study (in order to satisfy eligibility criteria			
Competency checklist		Tool: *competency checklist* Completed by: HCP When: on completion of training and if deemed necessary afterwards	Tool: *competency checklist* Completed by: HCP When: on completion of training and if deemed necessary afterwards	
SAE reporting			Including appropriateness of administration, proportionality, side effects, drug accountability, carer events	
Evaluation of training package				Tool: *qualitative interviewing* Completed by: carer and HCP When: post-bereavement
Assessment: impact on carer				
Self-efficacy		Tool: *QOLLTI-F* Completed by: carer When: at baseline before randomisation	Tool: *QOLLTI-F* Completed by: carer When: at the time the patient first needs as-needed SC medication and every 48 hours thereafter	Tool: *qualitative interviewing* Completed by: carer When: post-bereavement
Confidence			Tool: *carer diary* Completed by: carer When: after giving every injection	
Assessment: health economic outcomes				
Impact on carers		Tool: *CES* Completed by: carer When: baseline		Tool: *CES* Completed by: carer When: post-bereavement
Discrete Choice Experiment (DCE) attribute selection				Tool: *Qualitative interviewing* Completed by: carer When: post-bereavement

CES, Carer Experience Scale; CRF, Case Report Form; HCP, healthcare professional; MSAS-GDI, Memorial Symptom Assessment Score—General Distress Index; QOLLTI-F, Quality of Life in Life-Threatening Illness-Family Carer Version; SAE, serious adverse event.

Dyads were initially approached by their local district nurse or specialist palliative care team and were recruited by research nurses. Dynamic adaptive randomisation that stratified for recruitment centre and diagnosis (cancer/non-cancer) was carried out remotely by the recruiting research nurse using a secure online randomisation system.

Written informed consent was taken by a researcher at the time of approach, during the dying phase, at a time judged suitable by their HCP. Patients were approached first, followed by their choice of named carer. In cases where a patient did not have the capacity to consent, or lost capacity during the study, assent was sought from a personal consultee. In the qualitative work, informed consent was taken from carers at the time of interview and from HCPs on response of their agreement to take part (online supplemental file 1).

#### Intervention delivery

Intervention-group carers received training by community nurses (district nurses or specialist palliative care nurses) using a manualised training package.

#### Data collection

Data were collected through an assessment from a research nurse at baseline and follow-up (with the carer at 6–8 weeks post-bereavement) and via carer diaries. Baseline assessment included patient and carer demographic information, medical history, capacity assessment and current drug management.

##### Potential primary outcome measures for future definitive trial

Carers were asked to complete the Carer Experience Scale at baseline and follow-up and the Quality of Life in Life-Threatening Illness—Family Carer Version (QOLLTI-F) at baseline and every 48 hours after the first subcutaneous medication was administered for breakthrough symptoms. Carers completed the Family Memorial Symptom Assessment Score—General Distress Index (MSAS-GDI) at follow-up.

##### Carer diaries

Carer diaries were used to record incidences of breakthrough symptoms, including the symptom score before and after subcutaneous medication was administered and the time to symptom relief. In the intervention arm, diaries were also used to record carer confidence and whether or not HCP support was sought.

##### Embedded qualitative study

Carers were invited to participate in a qualitative interview 2–4 months post-bereavement, which included asking them to select attributes for a future discrete choice experiment. HCPs were also invited to take part in separate qualitative interviews to share their experiences. Interviews were audio-recorded and transcribed verbatim. Carer interviews were analysed using Interpretive Phenomenological Analysis and HCP interviews were analysed using the framework approach.^17 18^

##### Safety

The project contained a number of safety outcome measures at different stages of the clinical journey, including the risk assessment tool, competency checklist and significant event reporting. Significant event reporting included: the appropriateness of administration, proportionality, side effects, drug accountability and carer events. All adverse events (AEs) and serious adverse events (SAEs) were captured via the significant event form; SAEs were reported to the principal investigator (PI) and sponsor within 24 hours. As this was a study involving patients who were close to the end of their lives, death was an expected outcome. It was recorded and reported to the sponsor, but was not considered a SAE if, in the opinion of the PI, it was a natural conclusion to a patient’s life-limiting illness.

### Sample size

A fully justified sample size was not required; sample size was justified by estimating what sample size a future definitive RCT will need. Sim and Lewis recommend a sample size of approximately 50–55 to ensure robust estimates of the variance.^19^ Using estimates of dropouts, we predicted that we needed to approach 200 potential participants to achieve 100 randomised participants, with 50 completers (‘completer’ is defined as a dyad who completed all of the study measures from baseline to follow-up at 6–8 weeks post-bereavement).

### Statistical analysis

The primary analysis was concentrated on the feasibility metrics and adherence outcomes based on defined thresholds. There was limited preliminary analysis of intervention outcomes. Point and 95% CI estimates were calculated and used to estimate the variability and direction of effect to further inform the sample size calculation for a definitive study.

Summary statistics of all outcomes were used to inform the approximate models of analysis that would be used in a full trial. The results of the feasibility trial were used to inform the most appropriate analysis models (eg, the number of episodes in which as-needed medication was used and the proportion of participants who never required as-needed medication). A preliminary analysis of the outcomes was completed using an intention-to-treat approach. All analyses that were undertaken were prespecified in a statistical analysis plan that was written and agreed on before data collection was completed.

As this was a feasibility trial, there was no imputation of missing data. Missing data were considered as a criterion for assessing the suitability of measures. Descriptive statistics were produced for each of the outcome measures to evaluate the appropriateness of the measures for inclusion in a definitive RCT.

### Progression criteria

Recruitment of >30% of eligible dyads and retention of >40% of recruited dyads was assumed to indicate sufficient feasibility for progression to full trial. The expected recruitment target was ≈50 dyads.

To illuminate considerations for future definitive work further, we undertook a stepwise approach to interpret the feasibility study results. We applied the Shanyinde framework and summarised the positive findings and identified problems.^20^ Then, we used the A Process for Decision-making after Pilot and feasibility Trials framework to assess the identified problems and suggest solutions.^21^

### Patient and public involvement

Public contribution formed an integral part of the CARiAD trial and was part of each stage from development to dissemination. Our reporting of public contribution in the CARiAD trial is based on the GRIPP2 (Guidance for Reporting Involvement of Patients and the Public) checklist (version 2).^22^

A range of methods was used for public contribution at different stages throughout the study. These included the following:

- The initial decision to develop the CARiAD trial resulted from the Palliative and End of Life Care Priority Setting Partnership report, which incorporated the views of 1403 people across the UK, which placed great emphasis on empowerment of family carers and symptom management during the last days of life.^23^
- The study design was heavily informed by the work-up stages, which comprised two group consultations with carer groups (suggestions on consent mechanisms, drug safety, training and ongoing support were incorporated into the study design), one telephone consultation with a bereaved carer (JO’C, coauthor) who had given subcutaneous medications to her late husband and one telephone consultation with a nurse who had trained a carer to give subcutaneous medication to a relative and had supported them in doing so.
- The CARiAD trial had two public contributors as co-applicants to the study (BF and JO’C, both coauthors) who contributed to the submitted application, including providing the lay summary. Following this, one of the public contributors (JO’C, coauthor) attended and contributed significantly to the CARiAD Research Ethics Committee (REC) meeting.
- Trial materials, including the topic guides for the qualitative interviews, were produced with the input of the public contributors. Public contributors also took part in expert consensus workshops and formed part of the Trial Steering Committee.
- The public contributors strongly supported the study dissemination. One of the public contributor co-applicants (JO’C, coauthor) presented the results of the CARiAD study at a national palliative care conference.

The outcomes of public contribution in the early stages of the study included a better-designed study, particularly based on input during the study work-up phase. This added to, altered or confirmed a number of study design elements. Throughout the study, the study team was better informed through the sustained and active public contribution in meetings and developed a stronger sense of bereaved carers’ perspectives on the appropriateness of the approaches considered. In more practical ways, it was an enormous advantage to have the presence and participation of JO’C (coauthor) at the NHS REC meeting. This gave the REC a sense of the meaning of the study and how it may be perceived by bereaved carers and reassured them that the study intervention could be welcomed by carers. The public contribution improved the quality of a number of the public-facing documents, such as the qualitative topic guide and the study close-out documentation for carers. We identified no negative outcomes from public contribution.

## Results

### Eligibility, recruitment and retention of participants

**Figure 1:**
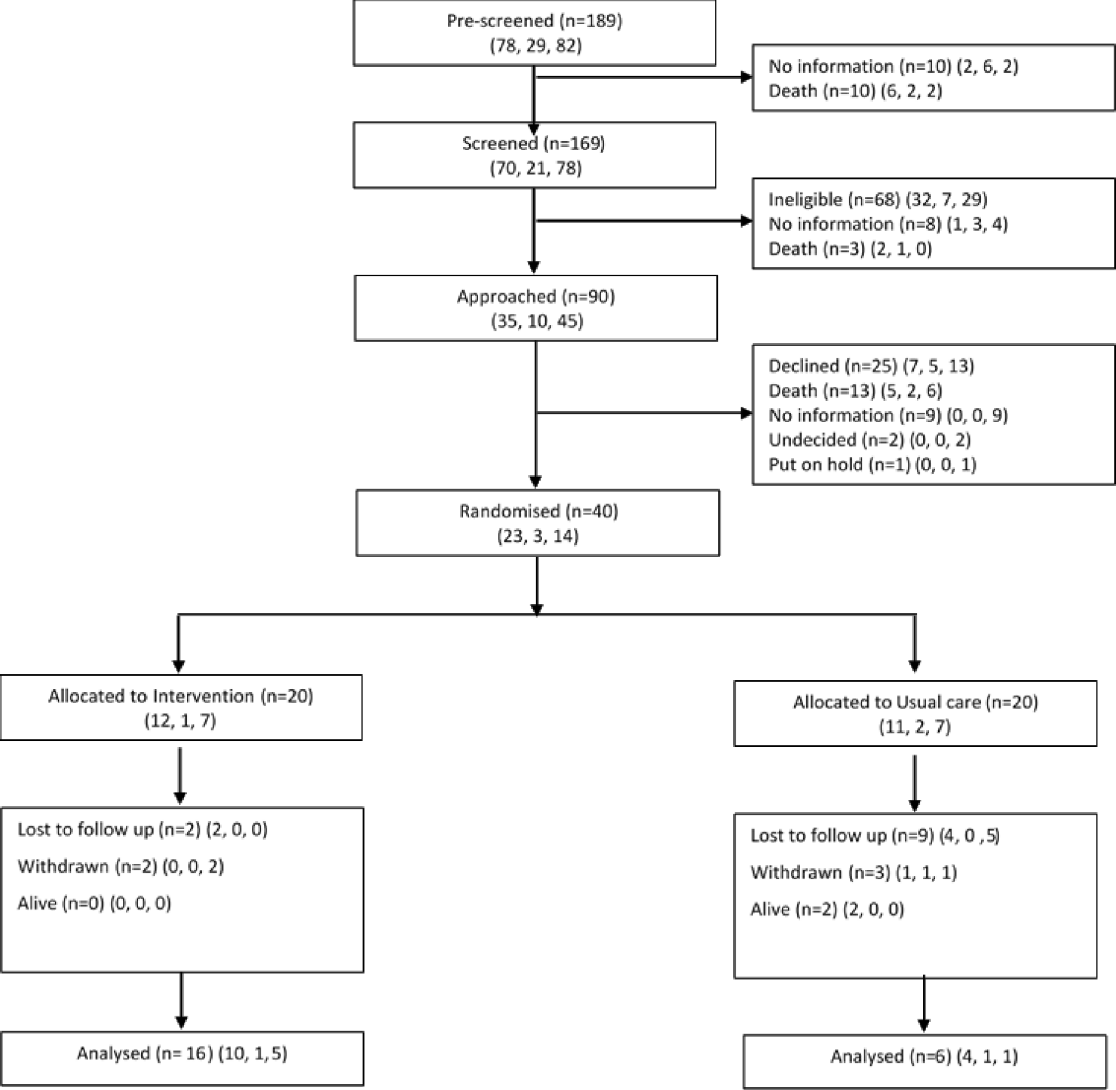
Consolidated Standards of Reporting Trials flow diagram for the CARiAD feasibility randomised controlled trial.

The study opened to recruitment in North Wales on 10 January 2018, in Vale of Glamorgan on 21 March 2018 and in Gloucestershire on 1 April 2018. Recruitment closed on 15 March 2019, allowing an average of 12 months per site from site opening. The flow of the participants through the trial is shown in figure 1.

#### Eligibility

There were 189 potential dyads identified across the three sites in which the patient was in the last weeks of their life and was likely to lose the oral route of medication administration. Of these, 169 were screened and 68 were ineligible. The main reasons for ineligibility were only paid or formal care being in place (18/68, 26.5%) and the patient not wishing to die at home (13/68, 19.1%). For 11 out of 169 dyads, eligibility was confirmed but no further information was provided (n=8) or the patient died before approach (n=3).

#### Recruitment and randomisation

A total of 101 dyads were eligible: 90 dyads were approached to participate and 40 dyads completed the baseline visit and were randomised (39.6% (40/101) of the eligible population, 44.4% (40/90) of those approached). This met the feasibility criterion of recruiting >30% of eligible dyads. 20 dyads were allocated to the intervention and 20 were allocated to usual care. The expected recruitment target (≈50) was not reached because fewer than expected participants were identified.

#### Retention

22 carers completed the follow-up visit (22/40, 55% of those randomised): 16 (16/20, 80%) from the intervention group and 6 (6/20, 30%) from the usual-care group. The feasibility criterion of >40% retention was, therefore, considered not met. It was possible to obtain some information from medical notes for a further three patients; therefore, partial data are available for 25 patients at follow-up.

In total, four (10%) dyads withdrew from the study. Three of these withdrew because they had been allocated to the usual-care group, and one withdrew because of concerns around possibly giving the last injection to the patient.

### Participant characteristics

#### Demographic information

Demographic information is detailed in table 2.

**Table 2:** Demographic information

	Patients	Carers
Age (mean)	68.3 years	56.6 years
Male	23/40 (57.5%)	5/40 (12.5%)
Female	17/40 (42.5%)	35/40 (87.5%)
Primary diagnosis: cancer	33/40 (82.5%)	
Primary diagnosis: non-cancer	7/40 (17.5%)	
Place of death: own home	18/25 (72%)	
Place of death: hospital	2/25 (8%)	
Place of death: hospice	5/25 (20%)	
Spouse or partner		20/40 (50%)
Living with patient		32/40 (80%)

#### Medication regimens

At baseline, 33 out of 40 (82.5%) patients had anticipatory prescribing in place. At the time of death, all patients (n=25) had anticipatory prescribing in place. At baseline, 27 out of 40 (67.5%) patients did not have a continuous subcutaneous infusion set up. There were 21 (21/25, 84%) patients who had a continuous subcutaneous infusion at the time of their death.

### Carer diary analyses

#### Returns

Three dyads from the usual-care group returned carer diaries, with 20 medication administration entries completed. 11 dyads from the intervention group returned carer diaries, with 147 medication administration entries completed.

#### Symptoms resulting in medication administration

Calculation of time to symptom relief depended on how complete the medication administration entries were; this was possible in 91.3% of entries in the intervention group and 75% of entries in the control group. In the intervention group, 88.8% of these had resolved to an acceptable level within 30 min compared with 26.7% in the usual-care group.

#### Time to symptom relief

The intervention group had a considerably shorter time to medication administration than the usual-care group: the median time to administer medication in the intervention group was 5 min and in the usual-care group was 105 minutes.

#### Carer confidence (intervention group)

Carers in the intervention group were asked how confident they felt about administering the medication. This was on a scale from 1 to 7, with a higher score indicating higher confidence. Of the 11 carers in the intervention group who returned carer diaries, 9 (81.8%) administered medications. Of the 131 instances in which the carer administered the medication, the confidence score was recorded in 128 (97.7%). For pain, nausea or vomiting and noisy breathing, the median score was 7, the highest level of confidence, and the median score was 6 for anxiety/restlessness. Although there is fluctuation in the scores over time, the overall trend shows an increase in carer confidence over time, with the final score being 6 or 7 for all of the carers.

#### HCP visits

HCP support was sought by carers in the intervention group in 24 out of 147 (16.3%) medication administration entries. These were not clustered at the beginning of the carer’s time in the study.

### Exploratory end points/primary outcomes for a future definitive trial

#### Quality of Life in Life-Threatening Illness—Family Carer Version

At baseline, only one carer did not complete the QOLLTI-F (35 carers completed 100% and four carers completed 70%–99%). It was intended that, after the baseline visit, to assess quality of life, the QOLLTI-F would be completed every 48 hours after the first injection for breakthrough symptoms. However, the QOLLTI-F was completed for only six dyads: four in the intervention group and two in the usual-care group. The high level of completion at baseline suggests that the measure is acceptable but that it is less feasible for carers to complete this independently and at regular intervals in this context (ie, while caring for someone in the last days of their life).

#### Family MSAS-GDI

All of the 22 carers who completed follow-up completed the Family MSAS-GDI. Of these, 19 (86.4%) carers completed all of the Family MSAS-GDI and 3 (13.6%) completed 75%–99% of the measure. From these results, at least 80% of the measure is completed; therefore, from this perspective, the Family MSAS-GDI can be considered as a primary outcome in a definitive trial.

### Acceptability and feasibility of the intervention

#### Carer experience

Qualitative interviews with 12 carers (intervention group, n=10; usual-care group, n=2) show that the intervention is acceptable to patients and carers, who found it helpful and reassuring. Key findings from carer interviews show that carers have a strong desire to fulfil patients wishes to have a home death and are glad of the opportunity to be able to help them have a good death and keep them symptom-free as much as possible. The intervention was shown to empower patients and carers by giving them greater control over the circumstances during the end of life. The QOLLTI-F was a source of confusion for carers. Carer concerns regarding euthanasia or hastening death can be relieved with training and reassurance from HCPs.

#### HCP experience

Interviews with 20 HCPs revealed that, although they mostly found the intervention to be acceptable in terms of patient and carer benefit, they also had concerns regarding the screening and selection of dyads. HCPs felt that it was important to be very careful about who was approached, which may be a result of concerns regarding carer coping ability and the risk for patients, but also of a desire for self-protection and concern about culpability should mistakes occur. HCPs reported time constraints owing to heavy workloads and were sometimes unable to prioritise recruitment and trial training over their other responsibilities. HCPs had a largely positive view of the intervention in terms of the dyads that they had supported and found that carers were also positive about the experience.

### Safety

In total, there were seven AEs and 20 SAEs reported. All AEs were reported according to the Bangor University standard operating procedure and, where necessary, further action was taken (eg, additional team training). All 20 SAEs were inpatient admissions to either a hospital or a hospice.

In total, there were 13 protocol deviations identified. Five of these were related to the recording of the risk assessment process prior to the approach. Two each were related to the sharing of patient-identifiable information with the central team, having more than one carer involved in the trial processes, and errors on the medication table (carer diary, intervention group). One each related to the mismatch between trial documentation and MACRO and declining the use of a continuous subcutaneous infusion despite clinical advice.

## Discussion

The CARiAD study explored the feasibility of testing the clinical effectiveness of the intervention of carer administration of as-needed medication for breakthrough symptoms in people dying at home in the UK, to inform the design of a future definitive trial. We concluded that the success of a future definitive trial is uncertain owing to equivocal results relating to trial feasibility, particularly that target recruitment was not reached and retention in the usual-care group was low. The context of the trial was not ideal: district nurses were seriously overstretched, unfamiliar with the research methods and possibly not in equipoise.

We found an apparent disparity between carers’ and HCPs’ readiness to consider the intervention: carers were uniformly enthusiastic and expressed a preference for the intervention arm while HCPs were more hesitant and felt unsure if they were in a position to support this in the current NHS context.

The intervention was shown to be acceptable, feasible and safe in the study population, and the overall recruitment and retention rate was above what was stated to be necessary for a definitive trial. Furthermore, noting that the intervention is already slowly spreading across more areas in the UK, it should be considered whether or not there is still an unanswered clinical effectiveness question. Our results lend some weight to this notion by demonstrating considerably shorter time to medication administration and faster symptom control in the intervention group, decreased need for HCP visits and almost universal positivity from carers, albeit in a small sample size. For this reason, a ‘common good’ argument could increasingly be defended.

Future work is clearly needed. This should include understanding the context of the areas in the UK where the practice has already been adopted, ascertaining wider public views on the intervention and understanding HCPs’ motivations/views on burden and risk and interface with the NHS context. Findings from both quantitative and qualitative data suggest that there is a need for consideration of the most appropriate outcome measures, including consideration of the expected impact of the intervention and where it can be best evidenced for effect. There should also be consideration of the need for national policy on the intervention. Owing to the small sample size and poor retention of the usual-care group, it may be that there are unanswered questions relating to the intervention that would be best studied in a trial in future; the work suggested above will help to ascertain if this is the case.

### Strengths and limitations

CARiAD is an exemplar of a well-conducted palliative care feasibility study and fulfilled its fundamental purpose of reducing research waste. The study illuminated numerous aspects of research related to last-days-of-life research: it illustrated both dying people’s and carers’ willingness to be randomised, strong and successful public contributor involvement at all stages and the value of rich qualitative findings in explaining the quantitative results. It used a structured approach to assess the outcome of such feasibility work. In addition, it generated a new UK training package and method for lay carer role extension that can be used in any further research or implementation and clarified limitations of community-based nursing care in three areas in the UK. It also illuminated areas for future work.

The key limitations are that (1) a series of key progression criteria were not met, (2) the differential recruitment to the control group was of particular concern and (3) overall, the limitations led to an equivocal decision about progression to a definitive RCT. The lack of a policy framework to underpin practice in the UK may have impeded the study in a number of ways; however, if such a policy framework existed, a randomised study would not have been appropriate. In terms of study work-up and design, the project did not benefit from considering a theory-driven approach to inform the rapid review or intervention design, nor an embedded process evaluation using a theoretical framework.

### Clinical implementation

In March 2020, whilst finalising the report to the funder, the CARiAD research team was asked to lead the development of all-Wales policy on the intervention, towards rapid implementation in the pandemic context. The funder gave permission for the draft final report to be released early to support adaptation for clinical use. The resulting policy and supporting materials were approved by Welsh Government and clinical implementation commenced shortly afterwards in north and mid-Wales^24^ .

Based on North Wales data (n=101 patients between April 2020 and December 2022), clinical outcomes are excellent: time to medication administration is short (median=10, IQR=17, 3–20), and carer confidence (in administering medication) is high. The intervention is safe and the supporting package is practical and welcomed by lay carers. Lay carers feel empowered and well-supported by HCPs. They highly value the opportunity to help their loved one die well in the place of their choice.

The UK-leading success in North Wales supported knowledge transfer on the intervention to many UK teams and the setting up of the National (UK) Community of Practice (CoP) for HCPs and managers interested or involved in the intervention. The CoP allows shared learning and is supported by a Future NHS platform workspace.

## Conclusion

Most people in the UK wish to be at home when they die. Carer administration of as-needed subcutaneous medication for common breakthrough symptoms shows much promise as a way to ensure timely symptom control and carer empowerment in this setting. As a future definitive trial of the intervention in the UK is highly unlikely to follow, future work should include robust evaluation of outcomes. Deeper understanding of the intervention and its place in care delivery for those dying at home will ensure that, in future, everyone who could benefit from the intervention is offered it.

## Supplementary material

10.1136/bmjopen-2024-084476 online supplemental file 1

## Data Availability

Data are available upon reasonable request.
