# Supplementary material for: Carer administration of as-needed subcutaneous medication for breakthrough symptoms in people dying at home: the CARiAD feasibility RCT
Source: BMJ Open. 2025 Jun 12;15(6):e084476. doi: 10.1136/bmjopen-2024-084476 (PMC12161397; doi:10.1136/bmjopen-2024-084476)
Supplement: online supplemental file 1 [file bmjopen-15-6-s001.pdf]

CARiAD Informed Consent Form - Patient participant

CARiAD – Carer Administration of as-needed sub-cutaneous medication for breakthrough symptoms in home-based dying patients

Participant Identification Number: | | | | |

Please initial box

1. I confirm that I have read, or have had read to me, the information sheet (v3 June 2018) for the CARiAD study and understand what is involved. I have had the opportunity to consider the information, ask questions and have had these answered satisfactorily.

2. I understand that I will be assigned to the new care or usual care group at random and that if I am in the usual care group the person caring for me will not be given the training required to administer as-needed sub-cutaneous medication and I will receive care as usual.

3. I understand that my participation is voluntary and that I am free to withdraw at any time without giving any reason and without the medical care or legal rights of myself or the person caring for me being affected. If I withdraw from the study the researchers will use the information I have provided up until that point, unless I indicate that I do not want them to.

4. I understand that my medical notes and data collected during the study will be looked at by individuals involved in the trial and may be accessed by regulatory authorities or the NHS Trust, where it is relevant to my taking part in this research. I understand that this data will be treated in the strictest confidence and give permission for these individuals to have access to this data.

5. I agree to my General Practitioner and other members of my healthcare team being informed of my participation in the study.

6. I understand that if I lose mental capacity, including the ability to communicate my wishes, then a consultee will be asked to advise on whether I should continue in the study.

7. I understand that if the researchers have any serious concerns about my health, safety or well-being, they have a duty to inform my GP or another appropriate professional.

8. I agree to take part in this study.

When completed: 1 for participant; 1 for researcher site file; 1 (original) to be kept in medical notes.

______________________	______________________	______________________
Name of Participant	Date	Signature
______________________	______________________	______________________
Name of Person taking consent	Date	Signature

When completed: 1 for participant; 1 for researcher site file; 1 (original) to be kept in medical notes.
