# Supplementary material for: Carer administration of as-needed subcutaneous medication for breakthrough symptoms in people dying at home: the CARiAD feasibility RCT
Source: BMJ Open. 2025 Jun 12;15(6):e084476. doi: 10.1136/bmjopen-2024-084476 (PMC12161397; doi:10.1136/bmjopen-2024-084476)
Supplement: Supplementary file 2 [file bmjopen-2024-084476.reviewer_comments.pdf]

PEER REVIEW HISTORY

BMJ Open publishes all reviews undertaken for accepted manuscripts. Reviewers are asked to complete a checklist review form and are provided with free text boxes to elaborate on their assessment. These free text comments are reproduced below.

ARTICLE DETAILS

Title (Provisional)

Carer administration of as-needed subcutaneous medication for breakthrough symptoms in people dying at home: the CARiAD feasibility RCT

Authors

Poolman, Marlise; Wright, Stella; Hendry, Annie; Goulden, Nia; Holmes, EA; Byrne, Anthony; Perkins, Paul; Hoare, Zoe; Nelson, Annmarie; Hiscock, Julia; Hughes, Dyfrig A; O'Connor, Julie; Foster, Betty; Reymond, Liz; Lewis, Penney; Wee, Bee; Roberts, Rossella; Parkinson, Anne; Roberts, Sian; Wilkinson, Clare

---

VERSION 1 - REVIEW

**Reviewer**	**1**
**Name**	**Mosoiu, Daniela**
**Affiliation**	**ICRC**
**Date**	**18-Mar-2024**
**COI**	**No**

Thank you for leading this research and for the accuracy of your report (including the limitations). I see this research as important and given courage to health care professionals from resource restrained settings where district nurses are scarce to empower family members to administer subcutaneously medication and by doing this to help patients receive a better control of their symptoms. We do this routinely in our home care setting the difference is that alongside the main caregiver who takes the lead in managing the drugs, we teach other family members who are available to help. My concern with you research design is related to the number of questionnaires the relatives have to fill in as I see them as a high burden for family members who care for dying relative. I think you have a much better insight into the experience of the family member with the qualitative study.

**Reviewer**	**2**
**Name**	**Nguyen, HQ**
**Affiliation**	**Kaiser Permanente Southern California**
**Date**	**17-Aug-2024**

**COI**	**None**

This is a very well written and thorough manuscript reporting on a mixed-methods feasibility RCT of training family caregivers to administer subcutaneous medications for breakthrough symptoms in people dying at home. The go/no criteria for moving forward with a full scale RCT were clearly articulated and the protocol was very detailed. It was unfortunate that the nurses responsible for training families on the medication administration either did not have favorable views of the approach and/or were disengaged from the process. Nonetheless, it was positive to see that time to symptom relief was shorter in the intervention group, albeit in a very small sample.

I was surprised to read, only at the end of the manuscript that partly why this trial would not have proceeded was a decision by the Welsh government for this intervention to be implemented immediately during the COVID-19 pandemic. This should be stated earlier in the manuscript.

**Reviewer**	**3**
**Name**	**de Graeff , Alexander**
**Affiliation**	**Utrecht University**
**Date**	**22-Dec-2024**
**COI**	**No competing interests**

I was very surprised to see that the authors previously published a paper in Health Technology Assessment (Poolman M, Roberts J, Wright S, Hendry A, Goulden N, Holmes EA, Byrne A, Perkins P, Hoare Z, Nelson A, Hiscock J, Hughes D, O'Connor J, Foster B, Reymond L, Healy S, Lewis P, Wee B, Johnstone R, Roberts R, Parkinson A, Roberts S, Wilkinson C. Carer administration of as-needed subcutaneous medication for breakthrough symptoms in people dying at home: the CARiAD feasibility RCT. Health Technol Assess. 2020 May;24(25):1-150, listed as ref 1 in the present paper, but not referred to in the text), that contained the majority of the results presented in the current paper. As far as I can see, only the results of the interviews with carers and health care professionals were not given in the previous paper. The abstracts are for 95% word by word the same. I find this unacceptable. The results of the present paper are largely redundant.

In view of the above, I have not reviewed the paper in detail.

That does not mean that the paper has no potential value. I think the additional findings of the current paper are valuable. Moreover, the paper gives also findings (strangely enough in the discussion, see Clinical implementation) about the implementation of the intervention. I do not know how these data have been collected.

My suggestion would be to completely rewrite the paper, not to focus on the feasibility of a new trial (of which they conclude that it is highly unlikely to follow) but on the additional (not previously published) data of the RCT and on the findings during the implementation. Research data on the value of administration of subcutaneous medication by lay carers are very relevant, especially in view of the reluctance of health care professionals (as shown in the paper), to involve lay carers in this respect.

VERSION 1 - AUTHOR RESPONSE

Comment	Response	Page
Reviewer 1		
Thank you for leading this research and for the accuracy of your report (including the limitations). I see this research as important and given courage to health care professionals from resource restrained settings where district nurses are scarce to empower family members to administer subcutaneously medication and by doing this to help patients receive a better control of their symptoms. We do this routinely in our home care setting the difference is that alongside the main caregiver who takes the lead in managing the drugs, we teach other family members who are available to help.	The authors would like to thank reviewer 1 for their kind and insightful comments.	
My concern with you research design is related to the number of questionnaires the relatives have to fill in as I see them as a high burden for family members who care for dying relative. I think you have a much better insight into the experience of the family member with the qualitative study.	The authors agree with reviewer 1 that this was burdensome for the carers. This was indeed explored in more depth during the qualitative work and will be included in more detail in a subsequent, qualitative publication.	
Reviewer 2		
This is a very well written and thorough manuscript reporting on a mixed-methods feasibility RCT of training family	The authors would like to thank reviewer 2 for their kind and insightful comments.	

caregivers to administer subcutaneous medications for breakthrough symptoms in people dying at home. The go/no criteria for moving forward with a full scale RCT were clearly articulated and the protocol was very detailed. It was unfortunate that the nurses responsible for training families on the medication administration either did not have favorable views of the approach and/or were disengaged from the process. Nonetheless, it was positive to see that time to symptom relief was shorter in the intervention group, albeit in a very small sample.		
I was surprised to read, only at the end of the manuscript that partly why this trial would not have proceeded was a decision by the Welsh government for this intervention to be implemented immediately during the COVID-19 pandemic. This should be stated earlier in the manuscript.	The authors apologise for the late mention of this. However, this section has now been removed at the request of the Editor.	
Reviewer 3		
I was very surprised to see that the authors previously published a paper in Health Technology Assessment (protocol paper) that contained the majority of the results presented in the current paper. As far as I can see, only the results of the interviews with carers and health care professionals were not given in the previous paper. The abstracts are for 95% word by word the same. I find this unacceptable. The results of the present paper are largely redundant.<br>In view of the above, I have	The authors would like to thank reviewer 3 for their comments and would like to take this opportunity to address concerns regarding dual publication.<br><br>The previous publication was expedited at our request by the NIHR in order to allow rapid clinical implementation of the intervention in the pandemic context. However, there are two reasons we feel it is important to pursue publication of these results in BMJ Open:	

not reviewed the paper in detail.	1. It is important for this information to reach a wider audience, especially clinicians, as it pertains to a new intervention which may reshape how we approach care at the very end of life at home in the UK.<br>2. We have also provided additional information about clinical implementation since the HTA monograph was published.<br><br>We hope that, given these exceptional circumstances and enduring relevance of the topic, the Editor will consider publication.	
That does not mean that the paper has no potential value. I think the additional findings of the current paper are valuable. Moreover, the paper gives also findings (strangely enough in the discussion, see Clinical implementation) about the implementation of the intervention. I do not know how these data have been collected.	The authors apologise for the late addition of the implementation section. This section has now been removed at the request of the Editor.	
My suggestion would be to completely rewrite the paper, not to focus on the feasibility of a new trial (of which they conclude that it is highly unlikely to follow) but on the additional (not previously published) data of the RCT and on the findings during the implementation. Research data on the value of administration of subcutaneous medication by lay carers are very relevant, especially in view of the	The authors would like to thank reviewer 3 for their very helpful advice and suggestions. The authors intend to publish subsequent publications regarding the clinical implementation work and the qualitative aspect of the study in further detail.	

reluctance of health care professionals (as shown in the paper), to involve lay carers in this respect.		
